# Present and future are getting confused: are we equipped to face the technological revolution?

**DOI:** 10.1186/s13052-023-01529-1

**Published:** 2023-12-12

**Authors:** Giorgio Perilongo, Rino Agostiniani, Giovanni Corsello, Gianluigi Marseglia, Maurizio Muraca, Anna Maria Staiano, Gianvincenzo Zuccotti, Eugenio Baraldi, For the Commission of Technology Innovation in Pediatrics, Italian Society of Pediatrics

**Affiliations:** 1 Department of Rare Diseases, University Hospital of Padua, Via Giustiniani 3, Padua, 35128 Italy; 2 Division of Pediatrics, Department of Woman’s & Child’s Health, University Hospital of Padua, Via Giustiniani 3, Padua, 35128 Italy; 3 Mother and Child Department, Division of Pediatric and Neonatology, Hospital Toscana Centro Pistoia, Pistoia, Italy; 4 Division of Pediatrics, “A.R.N.A.S.” Civic Hospital, Di Cristina Benfratelli, University of Palermo, Palermo, Italy; 5 Department of Clincal, Surgical, Diagnostic and Pediatric Science, IRCCS San Matteo, Pavia, Italy; 6 Division of Pediatrics, IRCCS San Matteo, Pavia, Italy; 7 Pediatric Research Institute “Città della Speranza”, Padua, Italy; 8 Department of Translational Medical Science, University “Federico II, Naples, Italy; 9 Pediatric Division, University “Federico II, Naples, Italy; 10 Department of Pediatric, Division of Pediatric, Unviersity of Milan, Milan, Italy; 11 Medical School, Unviersity of Milan, Milan, Italy; 12 Department of Woman’s and Child’s Health, University Hospital of Padua, Padua, Italy; 13 Division of Neonatology, University Hospital of Padua, Padua, Italy; 14 Pediatric Research Institute “Città della Speranza”, Padua, Italy

**Keywords:** Technology, Science, Infosphere, Academic Education

## Abstract

**Background:**

This is a commentary reporting the outcome of a workshop promoted by the Department of Woman’s and Child’s Health of the University of Padua (Italy) focused on the emerging issue of what seems to be the increasing agemone role of technology.

**Main body:**

Over the centuries, technology has always been at the service of science, with theoretical insights anticipating experimental proofs. Over the last decades, however, the situation has radically changed, due to several factors. Technology seems to be playing an agemone role. The present and notably the future generation of scientists have major challenges to face. They have to deal with the forces generated by the infosphera; to dominate the technology and to maintain the capacity of generating inquisitive, creative, ethical and spiritual thoughts capable of addressing new scientific hypotheses and projects directed to the individual and collective good. However, in this scenario, what seems more relevant is to focus all our efforts in preparing ourselves, first, and then the new generations to face these challenges. From this point of view, the academic institutions and the scientific societies, have a major responsibility to deal with.

**Conclusions:**

The academic ecosystem traditionally used to educate the new generation of professionals as well as, and most importantly, the cultural, the professional pathways presently used to form them need to be extensively revised. The time is running short and the stakes are high. The debate is open.

## Background

Technology’s trajectory has always been guided by scientific insights, making it a consequential outcome of scientific exploration. Over the last decades, however, the technologic advancements have been such that the technology in itself has acquired a power never experienced before, to the point that one may feel that the relationship between science and technology is going through a radical change. Several factors are contributing to generate this feeling.

## Main text

The first of these new factors is the vastness of the horizons that the recent advances of science and technology are opening in front of our eyes. Indeed, there is a common sense of being witnesses of a revolution, which for the entity of the impact, can be pared to the industrial revolution of the XVIII and IX century. The major difference between the two however is the speed with which this present revolution is occurring. Indeed, the velocity with which science is producing new knowledge, new insights into human life and with which technology is transforming knowledge into more and more powerful “tools” to further pushing science head, is something new in the human history. The velocity is such that present and future get confused.

The evidence of the tremendous power of technology has reached its peak during the last century, when on August 6 and 9, 1945, two atomic bombs were launched over the Japanese cities of Hiroshima and Nagasaki killing between 129.000 and 26.600 people. Nowadays, however technology, particularly thanks to the pervasive role of Artificial Intelligence [AI] and of the new biomedical and communication technologies, has shown a new and more destabilizing power; the power of manipulating the human mind, confusing the real world with the virtual one, and according to what should still be counted as science fiction (see “2001: a Space Odyssey, 1968, Director, Staniey KubricK”) overriding the man’s mind, guiding and influencing science and ultimately moving haed independently. Too much? Probably yes, but not entirely. Indeed, we all are under a negative pressure of forces that we, ourselves, have generated, which seem to be able to compromise some human faculties. The philosophers call “infosphere” the time we are leaving, being the “infosphere” the atmosphere in which we run our daily life dominated by the modern very powerful technologies, by the data they generate and by the outcome of digitalized big data processing [1]. The “infosphere” does not comprise only a series of inert tools ready to use, to increase the man’s power of dominating the world; it is not a passive force. Instead it is a force which can create, forge, manipulate our way of thinking, our way of behaving, our relationships, our organization and ultimately our minds if not the human life in itself [2]. We are referring to the power of AI and particularly of generative AI and, entering into the biological field, we are referring, to the CRISPY technique, which opened the way to the possibility of editing the human genome [3].

It is obvious. Nothing can be stopped! An intimate, intrinsic powerful impulse moves the research ahead regardless of any external factors; similarly, the technological advances are the intrinsic forces that generate new and more effective technologies; the technology generates itself. The ultimate result is that the power in the man’s hand to dominate the world is expected to increase and, at this point, exponentially. Actually, this consideration should not surprise; in fact that was the mandate that God gave to Adam (Gen 1, 26). Thus, the question is not how far science will develop and where technology will bring the human’s power; instead the question is: will the man be capable of handling this power? This is an old question that the man has always asked to himself. It comes to the mind the myth of Atlant who was condemned to hold the entire celestial sphere. Is there the risk that the weight of what the man is asked to support can overweight his own force? Actually even for this question, it is not worth looking for a definitive answer.

The present and notably the future generation of scientists have major challenges to face. They have to deal with the forces generated by the “infosphere”. They have to dominate the technology, to make it a sort of co-pilot of many human activities, assuming a “mature” relationship with it. They have to maintain the capacity of generating inquisitive, creative, ethical and spiritual thoughts capable of addressing new scientific hypotheses and projects directed to the individual and collective good; capable of discerning the actual needs, the actual problems to address in order to minimize the risk of embracing passively any new technologic tools become available. The huge heritage of the European humanistic culture as well as the deep respect of the methodology to run research could be fundamental for this purpose.

Rules elaborated by national and international regulatory bodies indeed will help tremendously. The forces pushing technologies ahead considering the worldwide dimension of the problem require adequate regulatory response by supranational authorities. No single individual nor even any single nation will be able to elaborate rules capable of constraining and possibly controlling those forces. For this reason, “The Artificial Intelligence Act,” officially approved by the European Parliament on June 14 2023, represents one of the most relevant international initiative. It aims to provide rules, which according to the European rights and values should regulate the use of AI [4]. It is predicted that this treats will have ultimately an impact also on our daily practice.

In this context however, we want to drown the attention on how preparing ourselves, first, and then the new generation to face these challenges. From this point of view, the academic institutions and the scientific societies, have a major responsibility to deal with. In this perspective and specifically focusing in the medical and in this case in the pediatric field, urge all of us to envision multidisciplinary education programmes that equip pediatricians with a diverse skill set, preparing them to navigate complex challenges holistically. Thus, it seems relevant to corroborate the conventional educational and professional medical curriculum with knowledge regarding the modern tools that the technology made them available [e.g. how they work; the potentials and the limitations, the risks that their acritical use may imply] to practice modern medicine.

## Conclusions

The velocity with which science and technology are progressing is overriding the cultural, the professional, and the actual educational and research experiences of many professionals living the Academy in these days. Most likely, also the academic ecosystem traditionally used to educate the new generations of professionals as well as, and most importantly, the cultural, the professional pathways presently used to form them are obsolete. Any single professionals involved in mentoring in one way of the other the new generations of professionals and any educational and research academic institution have to address this fact and promptly react. Needless, to say that only a combined multi-professional, multicultural approach and renewed partnerships can allow elaborating effect solutions. The time is running short and the stakes are high. The debate is open.

## Data Availability

The manuscript does not contain any data and materials; thus the related statement is. “Not applicable”.
